# Nrf2 as a Therapeutic Target in the Resistance to Targeted Therapies in Melanoma

**DOI:** 10.3390/antiox12061313

**Published:** 2023-06-20

**Authors:** Marie Angèle Cucci, Margherita Grattarola, Chiara Monge, Antonella Roetto, Giuseppina Barrera, Emilia Caputo, Chiara Dianzani, Stefania Pizzimenti

**Affiliations:** 1 Department of Clinical and Biological Science, University of Turin, Corso Raffaello 30, 10125 Turin, Italy; marieangele.cucci@unito.it (M.A.C.); margherita.grattarola@unito.it (M.G.); giuseppina.barrera@unito.it (G.B.); 2 Department of Scienza e Tecnologia del Farmaco, University of Turin, Via Pietro Giuria 9, 10125 Turin, Italy; chiara.monge@unito.it (C.M.); chiara.dianzani@unito.it (C.D.); 3 Department of Clinical and Biological Sciences—San Luigi Gonzaga Hospital, University of Turin, Regione Gonzole 10, 10043 Orbassano, Turin, Italy; antonella.roetto@unito.it; 4 Institute of Genetics and Biophysics-IGB-CNR, “A. Buzzati-Traverso”, Via Pietro Castellino 111, 80131 Naples, Italy; emilia.caputo@igb.cnr.it

**Keywords:** melanoma, targeted therapy resistance, BRAFi/MEKi, dabrafenib, trametinib, Nrf2, YAP, DUB3, D4M cell line, A375 cell line

## Abstract

The use of specific inhibitors towards mutant BRAF (BRAFi) and MEK (MEKi) in BRAF-mutated patients has significantly improved progression-free and overall survival of metastatic melanoma patients. Nevertheless, half of the patients still develop resistance within the first year of therapy. Therefore, understanding the mechanisms of BRAFi/MEKi-acquired resistance has become a priority for researchers. Among others, oxidative stress-related mechanisms have emerged as a major force. The aim of this study was to evaluate the contribution of Nrf2, the master regulator of the cytoprotective and antioxidant response, in the BRAFi/MEKi acquired resistance of melanoma. Moreover, we investigated the mechanisms of its activity regulation and the possible cooperation with the oncogene YAP, which is also involved in chemoresistance. Taking advantage of established in vitro melanoma models resistant to BRAFi, MEKi, or dual resistance to BRAFi/MEKi, we demonstrated that Nrf2 was upregulated in melanoma cells resistant to targeted therapy at the post-translational level and that the deubiquitinase DUB3 participated in the control of the Nrf2 protein stability. Furthermore, we found that Nrf2 controlled the expression of YAP. Importantly, the inhibition of Nrf2, directly or through inhibition of DUB3, reverted the resistance to targeted therapies.

## 1. Introduction

Cutaneous melanoma is the 17th most common cancer worldwide, with 324,635 new cases and 57,043 deaths in 2020 [1]. The worldwide incidence of melanoma has risen rapidly in the last 50 years [2]. It accounts for only about 1% of cutaneous malignancies, but it is the most lethal form of skin cancer [3]. Although at early stages, melanoma has a very high survival rate (5-year survival > 99%); however, the advanced disease is poorly responsive to treatment based on conventional chemotherapy or radiotherapy, resulting in a 5-year survival rate of only 15% [4]. Fortunately, since 2011 new targeted treatments and immunotherapy [5] have led to a significant increase in survival. The 5-year relative survival for advanced stages of melanoma has doubled over the past decade, from 15–16% for patients diagnosed during 2004–2011 to 39.3% during 2016–2018 [4]. Mutational status of the cytoplasmic serine/threonine kinase mutant v-Raf murine sarcoma viral oncogene homolog B1 (BRAF), belonging to the mitogen-activated protein kinase (MAPK) signaling pathway, is the main criterion adopted to decide the best therapeutic option for advanced patients. Indeed, in the presence of BRAF mutation, both anti-BRAF targeted therapies, such as dabrafenib or vemurafenib, and checkpoint inhibitors, such as the anti-programmed death 1 (PD-1) blockers nivolumab and pembrolizumab, or the cytotoxic T-lymphocyte antigen 4 (CTLA-4) blocker ipilimumab can be used, while in BRAF-wild type melanoma patients, only immunotherapy can be delivered [6]. Activating mutations of BRAF mutations are present in ~50% of all melanoma cases and involve a substitution of the valine at position 600 (V600). More than 90% of those have a valine–glutamic acid substitution (V600E), followed by the less frequent missense mutations V600K, V600R, and V600D [7]. Treatment with BRAF inhibitor (BRAFi), selectively targeting the BRAF V600 genetic alteration, results in high response rates, further increased with the simultaneous addition of an inhibitor of mitogen-activated protein kinase (MEK), the BRAF downstream effector. Several combinations of BRAFi/MEKi are currently available, such as dabrafenib/trametinib, vemurafenib/cobimetinib, and the most recently introduced encorafenib/binimetinib [6]. When comparing BRAFi/MEKi combination therapy with BRAFi alone, increases in progression-free survival (PFS) and overall survival (OS) have been proven [8,9,10].

Despite significant improvements with the BRAFi/MEKi combination therapy, acquired resistance frequently develops within one year from the treatments, determining a real limit to their efficacy. Thus, there is an intense effort to understand the mechanisms of resistance to BRAFi/MEKi, with the aim of identifying new therapeutic targets. Both genetic and epigenetic mechanisms have been associated with the acquired resistance, mainly resulting in the reactivation of MAPK and, to a lesser extent, phosphatidylinositol 3-kinase (PI3K) and protein kinase B (AKT) pathways [11]. Recently, oxidative stress has become an emerging factor involved in drug resistance. Indeed, redox biology, which refers to the study of biochemical processes guided by reactive oxygen/nitrogen species (ROS and RNS) and antioxidants, plays a central role in melanoma and other solid cancers [12,13]. When ROS/RNS are produced in excess with respect to the antioxidant defenses, oxidative stress occurs. One of the main proteins involved in regulating antioxidant response is the Nuclear factor E2-related factor 2 (Nrf2), a transcription factor that has been found to play a pivotal role in several types of cancer [14], including melanoma [15,16]. In physiological conditions, Nrf2 is located in the cytosol, where it is coupled to its inhibitor Kelch-like ECH-associated protein (KEAP1), resulting in its degradation via ubiquitination and proteasomal-dependent proteolysis. Under oxidative stress, cysteine residues of KEAP1 are oxidized, leading to a conformational alteration. Thus, Nrf2 is released, and it can translocate into the nucleus where it binds to the Antioxidant Response Elements (ARE) sequences located in the promoter of specific antioxidant and cytoprotective genes, including Heme oxygenase-1 (HO-1) and numerous genes related to glutathione (GSH) metabolism, such as γ-glutamate cysteine ligase and glutathione-S-transferase A4 (GSTA4) [14]. Nrf2 can be a double-edged sword in several types of cancers, including melanoma [16,17]. Indeed, a cytoprotective role for Nrf2 has been recognized in the early stages of malignant transformation due to its ability to inhibit tumor growth, detoxify carcinogens, and protect cells from oxidative stress damage. However, an opposite role of Nrf2 in the advanced stages of the cancer disease has been highlighted since aberrant activations of Nrf2 are associated with poor prognosis, induction of pro-survival genes, promotion of cancer cell proliferation by metabolic reprogramming, and enhancement of the self-renewal capacity of cancer stem cells. Interestingly, Nrf2 is involved in resistance to chemotherapeutic drugs and radiation treatments [16,17,18,19]. In melanoma, immunohistochemistry studies revealed that Nrf2 protein expression correlated with deeper Breslow, invasive phenotype, nodular growth, and worse survival [20]. Moreover, Nrf2 inhibition enhanced melanoma sensitivity to conventional drugs, such as cisplatin (CDDP), dacarbazine (DTIC), or temozolomide (TMZ) [21,22,23]. Interestingly, Nrf2 inhibition can also overcome melanoma radioresistance [18].

More than 2000 genes can be directly or indirectly regulated by Nrf2. The majority are involved in maintaining redox balance; however, others contribute to the balance among metabolic pathways, thus apparently unrelated to oxidative stress [24]. Recently our group identified crosstalk between Nrf2 and Yes-associated protein (YAP) in bladder [25] and pancreatic cancer cells [26], further extending the complex Nrf2 network. This transcriptional coactivator is a key component of the Hippo tumor suppressor pathway [27]. When the Hippo pathway is on, YAP phosphorylation occurs in precise serine residues (Ser 127, Ser 381), leading to its inactivation through cytoplasmic retention or YAP ubiquitination and subsequent proteasomal degradation. Conversely, the inactivity of the Hippo pathway maintains YAP under unphosphorylated status, thus enabling it to translocate into the nucleus, bind transcription factors mainly belonging to the TEA domain family members (TEAD), and induce the expression of several downstream target genes, such as Survivin and Forkhead Box M1 (FoxM1), involved in cell number and organ size through regulation of cell proliferation and cell survival [28,29]. YAP can also contribute to the maintenance of the cellular antioxidant status since it can interact with the Forkhead Box O1 (FoxO1) transcription factor, which activates the expression of catalase and manganese superoxide dismutase (MnSOD), two well-known antioxidant genes [30]. The oncoprotein YAP is frequently activated in cancer, and it is essential for both initial phases and late stages during the progression of most solid tumors [27]. In melanoma, it has been shown to promote cell invasion and spontaneous metastasis [31]. Several mechanisms lead to Nrf2 activation in cancer [32]. They include gain-of-function mutations in the Nrf2 gene, loss-of-function mutations in the KEAP1 gene, or upregulation of Nrf2 gene expression via oncogenic signaling. Epigenetic mechanisms have also been observed, such as hypermethylation of the KEAP1 promoter, eliciting the reduction of KEAP1 gene transcription and the accumulation of the Nrf2 protein, or abnormal Nrf2 or KEAP1 mRNA splicing. Moreover, post-translation mechanisms have been elucidated: i.e., the p62 Nrf2-KEAP1 disruptor leading to autophagic degradation of KEAP1 and dysregulation of microRNA targeting Nrf2 or KEAP1 [32]. In melanoma, a frameshift KEAP1 mutation, leading to the aberrant activation of NRF2, was found to be associated with an increase in chemoresistance to CDDP or DTIC [21]. However, recently, in a large cohort of melanoma tumor samples with different degrees of malignancy, it was demonstrated that there was no correlation between immunostaining of the Nrf2 protein and mRNA expression levels [33]. Thus, it is very likely that post-translational mechanisms can account for the Nrf2 activation in melanoma [34]. Recently, the pivotal role of deubiquitinases (DUBs) as druggable targets in melanoma has been reported [35,36]. The specific DUB3 has been found to stabilize Nrf2, conferring chemotherapy resistance in cancer [26,31]. To the best of our knowledge, no data are available about the role of DUB3 on melanoma progression.

In this study, we examined the contribution of Nrf2 in the BRAFi/MEKi-acquired resistance in melanoma cells resistant to DAB (single resistance to BRAFi), TRA (single resistance to MEKi), and DAB plus TRA (dual resistance to BRAFi/MEKi). Moreover, the mechanisms of its activity regulation and the possible Nrf2 cooperation with YAP were investigated.

## 2. Materials and Methods

### 2.1. Materials

Dimethyl sulfoxide (DMSO), 3-(4,5-dimethyl thiazol-2-yl)-2,5-diphenyltetrazolium bromide (MTT), agar, ultra-low 96-well plates, crystal violet, methanol, acetic acid, M199 medium, heparin, and bovine brain extract were from Merk Life Science S.r.l. (Milan, Italy). Dulbecco’s Modified Eagle Medium (DMEM) high glucose, Fetal Bovine Serum (FBS), penicillin, streptomycin, and trypsin were from Euroclone (Pero, Milan, Italy). Dabrafenib and trametinib were from Aurogene (Rome, Italy). Transwell Boyden Chamber, Matrigel, and fluorescein isothiocyanate (FITC) Annexin V Apoptosis Detection Kit (Cat. No. 556547) were from BD Biosciences (Milan, Italy). Chemiluminescence reagents (Western Lightning™ Chemiluminescence Reagent Plus ECL, PerkinElmer NEL105001EA) were from Life Sciences & Diagnostics (Milan, Italy). Antibodies used were as follows: Nrf2 (sc-365949), HO-1 (F-4 sc-390991), Keap1 (sc-33569), YAP (sc-376830), Survivin (D-8, sc-17779), FoxM1 (sc-271746), and β-actin (sc-47778) were from Santa Cruz Biotechnology, Inc. (Heidelberg, Germany); DUB3 (WHO 377630M1-100UG) was from Merk Life Science S.r.l. The following TaqMan^®^ Gene Expression Assays (Thermo Fisher Scientific, Waltham, MA, USA) were used: Hs00975960_m1 for the human Nrf2 gene and Hs00245445_m1 for both the human and murine Abl genes, respectively. Primer sequences (5′ to 3′) used to analyze murine Nrf2 expression were the following: mNrf2 Fw: 5′ACTTGGAGTTGCCACCG3′; mNrf2 Rw: 5′TTCTCCTGTTCCTTCTGGAG3′. SiRNA targeting Nrf2 (sc-143189) or DUB3 (sc-143189) and Transfection Medium (sc-36868) were from Santa Cruz Biotechnology, Inc. 

### 2.2. Cells, Culture Conditions 

The Dartmouth Murine Mutant Malignant Melanoma (D4M) cells were kindly provided by Dr. Mullis (Department of Medicine, Norris Cotton Cancer Centre, Geisel School of Medicine at Dartmouth, Lebanon, NH, USA). This engineered murine cell line, established from the conditional mouse model of metastatic melanoma, harbors the BrafV600E mutation, and it has been found able to recapitulate the features of the human disease [37]. D4M subclones resistant to BRAFi/MEKi were generated in our laboratories, as described in the following section. Human melanoma cell line A375, sensitive and resistant to 200 nM DAB, were obtained from Prof. Caputo (Institute of Genetics and Biophysics-IGB, A. Buzzati-Traverso, CNR, Naples, Italy), and they were previously characterized [38]. All the cell lines used in this study were cultured in DMEM high glucose media, supplemented with 10% FBS, 100 units/mL penicillin, and 100 μg/mL streptomycin in a 5% CO_2_, 37 °C incubator.

Human umbilical vein endothelial cells (HUVECs) were isolated from human umbilical veins by trypsin treatment (1%) and cultured in M199 medium with the addition of 20% FBS, 100 U/mL penicillin, 100 μg/mL streptomycin, 5 UI/mL heparin, 12 μg/mL bovine brain extract, and 200 mM glutamine. HUVECs were grown to confluence in flasks and used at the 2nd–5th passages. The use of HUVECs was approved by the Ethics Committee of the “Presidio Ospedaliero Martini” of Turin (Italy) and conducted in accordance with the Declaration of Helsinki.

### 2.3. Generation of D4M Subclones Resistant to Dabrafenib, Trametinib, or Dual Resistance to Dabrafenib/Trametinib

To generate resistance to targeted therapy, D4M sensitive cells were continuously exposed to increasing concentrations of the BRAFi dabrafenib (D4M_DABres), MEKi trametinib (D4M_TRAres), or BRAFi dabrafenib plus MEKi trametinib (D4M_(D+T)res) up to 1.5 μM DAB and 36 nM TRA, for almost nine months. The maximum concentrations reached for each drug correspond to the peak plasma concentration reported for patients treated with the drugs [39,40]. As references, cells were also treated with DMSO, the solvent of drugs (D4M_DMSO), or untreated, then used as sensitive control cells (D4M_SENS). Since the resistance to targeted therapies in melanoma can be reversible and adaptive [41], the resistant cells were kept under drug-selective pressure. We suspended the treatment one week before to perform cellular assays.

### 2.4. MTT Assay 

Cell viability was assessed using the MTT assay, as previously described [26]. D4M cells (1500 cells/well) or A375 cells (5000–8000 cells/well) were seeded in 100 μL of the serum-supplemented medium onto a 96-well plate, starting with the drug treatments the next day. Untreated cells were used as a control. After treatments, the viability was assessed by adding 0.5 mg/mL MTT to control and treated cells for 2 h. Afterward, the medium was removed, 100 μL of DMSO was added, and the absorbance was recorded at 530 nm in a microplate ELISA reader. 

### 2.5. Anchorage-Independent Growth Assay

To assess the ability of cells to grow independently on a solid surface, two types of assays were performed. 

#### 2.5.1. Sphere Formation Assay

For the sphere formation assay, D4M cell lines (5000–8000 cells/well) were plated in ultra-low 96-well plates and resuspended in DMEM complete medium. For each D4M cell subclone, 8 wells were plated, and the experiment was repeated three times. After 72 h, digital images of wells were captured under phase-contrast microscopy observation in at least 4 random fields per well. The diameter of each sphere was calculated by using the open software platform ImageJ Version 1.54d (National Institutes of Health (NIH), Bethesda, MD, USA). Moreover, we calculated the number of spheres with a diameter greater than 50 μm. 

#### 2.5.2. Soft Agar Colony Formation Assay

For the soft agar assay, cells (10,000 cells/well) were suspended in 1.5 mL DMEM supplemented with 10% FCS and 0.4% agar. The suspension was poured over 1.5 mL pre-solidified 0.8% agar in 6-well plates, and the plates were incubated at 37 °C with 5% CO_2_ atmosphere saturation. Cells were cultivated for 3 weeks, and the growth medium on top of the agarose layer was replaced twice a week. Colonies were photographed using an inverted phase contrast microscope at 10× magnification and counted using image analysis software.

### 2.6. Cell Apoptosis

Apoptosis was assessed with Annexin V/propidium iodide (PI) assay, followed by flow cytometry analysis. D4M cell lines were seeded in 6-well plates (250,000 cells/well). After 24 h of the treatments, adherent and non-adherent cells were collected and processed as previously described [19], stained with Annexin V, conjugated to the fluorescein isothiocyanate (FITC) dye, and PI, according to the manufacturer’s protocol. Cells were analyzed using a FACScan cytometer Accuri C6 (Becton Dickinson Italia, Milan, Italy).

### 2.7. Cell Invasion Assay 

Preliminary experiments were performed to verify that the treatments were not toxic to cells. In particular, D4M cell lines were seeded into 96-well plates and treated with BRAFi/MEKi at the indicated concentrations for 6 h. Cell viability was analyzed with a crystal violet assay, as previously described [42]. Briefly, after treatments, D4M cells were fixed and stained with a solution of 80% crystal violet and 20% methanol. Then, 30% *v*/*v* acetic acid was added to induce a complete dissolution of the crystal violet. Absorbance was recorded at 595 nm in a microplate ELISA reader. After confirming the absence of cytotoxic effects at such an early time, D4M cell lines (1000 cells/well) were plated onto the apical side of a 50 μg/mL Matrigel-coated filter (8.2 mm diameter and 0.5 μm pore size) in a serum-free medium with or without the drugs under study. A medium containing FCS 20% was placed in the basolateral chamber as a chemoattractant. The chamber was incubated at 37 °C under 5% CO_2_. After 6 h, the cells on the apical side were wiped off. Cells on the bottom of the filter were stained with crystal violet, as described above, and counted (five fields of each triplicate filter) with an inverted microscope (magnification 40×). 

### 2.8. Angiogenesis Assay

In the tube formation assay, HUVECs were seeded onto 48-well plates (50,000 cells/well) previously coated with 75 μL growth factor-reduced Matrigel. At the same time, D4M cell lines were seeded into 96-well plates and treated with BRAFi/MEKi at the indicated concentrations for 6 h. Then, the conditioned media were collected and added to the HUVECs. The morphology of the capillary-like structures formed by the HUVECs was analyzed after 6 h of culture as previously described [42]. Briefly, tube formation was analyzed with an imaging system (Image Pro Plus Software for micro-imaging, Media Cybernetics, version 5.0, Bethesda, MD, USA) and evaluated by counting the total number of tubes. HUVECs that had received conditioned media from D4M_SENS cell culture were normalized to 100%.

### 2.9. Measurement of the Cell Redox Status

The oxidative stress level in D4M cell lines (200,000 cells/well) was measured with the 2′-7′-dichlorodihydrofluorescein diacetate (DCF-DA) fluorogenic probe as previously described [26]. Briefly, D4M cells lines were incubated for 30 min with DCF-DA, and the amount of fluorescent product 2′,7′-dichlorofluorescein (DCF) was assessed by cytofluorimetric analysis (Becton Dickinson Accuri, Franklin Lakes, NJ, USA).

### 2.10. GSH Content

GSH contents in D4M cell lines were assessed by determining non-protein sulphydryl contents with the Ellman’s method, as previously reported [43]. 

### 2.11. Protein Extraction and Western Blot Analysis 

Protein extraction and Western blot analysis were performed as previously described [25]. Antibodies used are listed in “Section 2.1”. The detection of the bands was carried out after the chemiluminescence reaction with ECL and visualized using a Bio-Rad visualizer (Bio-Rad Molecular Imager, ChemiDoc XRS+, Bio-Rad Laboratories, Hercules, CA, USA).

### 2.12. Quantitative Reverse Transcription Polymerase Chain Reaction (qRT-PCR)

RNA extraction, reverse transcription, cDNA synthesis, and quantitative real-time PCR reactions were performed as previously described [26]. Primers used to analyze human Nrf2 expression and the housekeeping Abelson (Abl) human and murine genes are listed in “Section 2.1”. 

### 2.13. Cell Transfection with siRNA against Nrf2 or DUB3 

Cellular transfection with siRNA targeting Nrf2 or DUB3 was performed with a protocol indicated by the manufacturer. Details about the specific siRNAs used are listed in “Section 2.1”. Cells were seeded onto 6-well tissue culture plates in the culture medium containing serum but not antibiotics. After 24 h, siRNA and the Transfection Reagent were diluted in siRNA Transfection Medium and incubated for 15 min at room temperature to allow the complexation between the siRNA and the Transfection Reagent. Afterward, siRNA transfection was carried out in the culture medium by adding complexes dropwise onto the cells. 

### 2.14. Statistics

The statistical significance was evaluated by one-way ANOVA followed by the multiple comparison post-test Bonferroni (GraphPad InStat software (San Diego, CA, USA)). We considered statistically significant values of *p* ≤ 0.05.

## 3. Results

### 3.1. Generation and Characterization of D4M Cell Lines Resistant to Dabrafenib, Trametinib, or Dual Resistance to Dabrafenib/Trametinib

#### 3.1.1. Cell Viability

D4M sensitive cells were continuously exposed to increasing concentrations of the BRAFi dabrafenib (D4M_DABres), MEKi trametinib (D4M_TRAres), or BRAFi dabrafenib plus MEKi trametinib (D4M_(D+T)res) up to 1.5 μM DAB and 36 nM TRA, for almost 9 months. The maximum concentrations reached for each drug correspond to the peak plasma concentration reported for patients treated with the drugs as described in Materials and Methods. As references, cells were also treated with DMSO, the solvent of drugs (D4M_DMSO), or untreated, then used as sensitive control cells (D4M_SENS). The obtained resistant subclones were then exposed to the drugs. 

**Figure 1:**
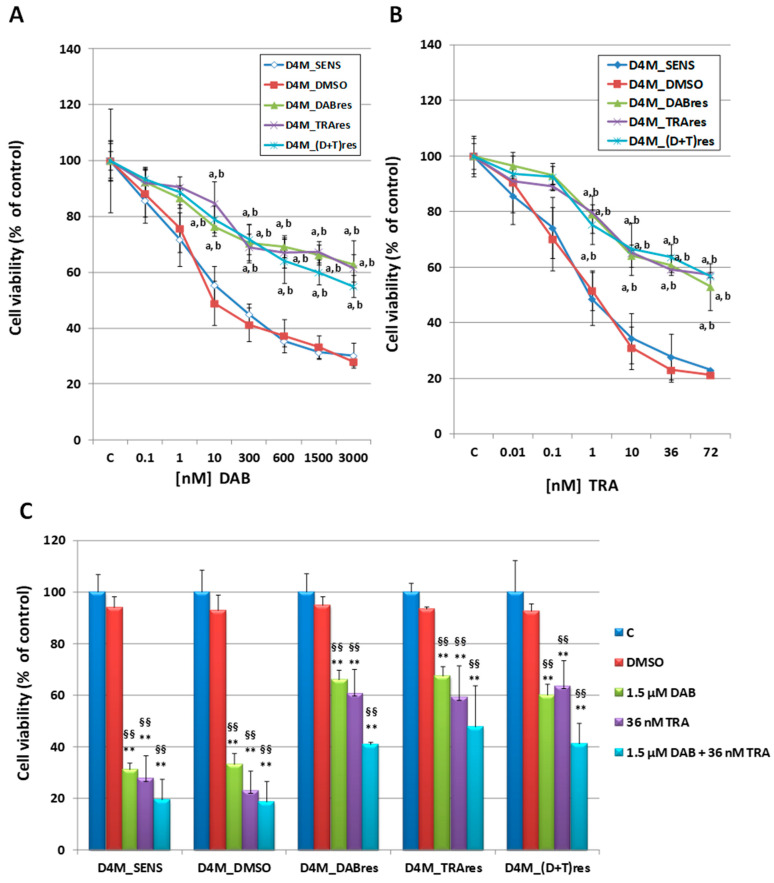
Viability (MTT assay) in D4M_SENS, D4M_DMSO, D4M_DABres, D4M_TRAres, and D4M_(D+T)res untreated or exposed to dabrafenib (DAB) (**A**), or trametinib (TRA) (**B**) at the indicated concentrations 72 h after the treatment. Results are expressed as a percent of respective untreated control (C) and are the mean ± SD of six separate experiments. a: *p* < 0.01 vs. D4M_SENS; b: *p* < 0.01 vs. D4M_DMSO. (**C**) Viability (MTT assay) in D4M_SENS, D4M_DMSO, D4M_DABres, D4M_TRAres, and D4M_(D+T)res untreated or exposed to DMSO (dilution 1/1000), 1.5 μM DAB, 36 nM TRA, and 1.5 μM DAB plus 36 nM TRA combined treatments for 72 h. Results are expressed as a percent of respective untreated control (C) and are the mean ± SD of six separate experiments. ** *p* < 0.01 vs. D4M_SENS; §§ *p* < 0.01 vs. D4M_DMSO.

MTT assays, performed after 72 h of drug exposition with increasing concentrations of dabrafenib (Figure 1A) or trametinib (Figure 1B), confirmed that all three resistant subclones were significantly less sensitive to the drug treatments with respect to both D4M_SENS or D4M_DMSO cells, which showed a similar viability inhibition. Interestingly, D4M_TRAres were also resistant to the treatment with DAB, as well as D4M_DABres were also resistant to the treatment with TRA. The dual resistant cell line D4M_(D+T)res was resistant to the single treatments (Figure 1A,B), as well as to the 1.5 μM DAB plus 36 nM TRA combined treatments (Figure 1C). Although viability after combined treatment was lower, however, any significant difference with respect to single treatments was observed.

#### 3.1.2. Anchorage-Independent Cell Growth

**Figure 2:**
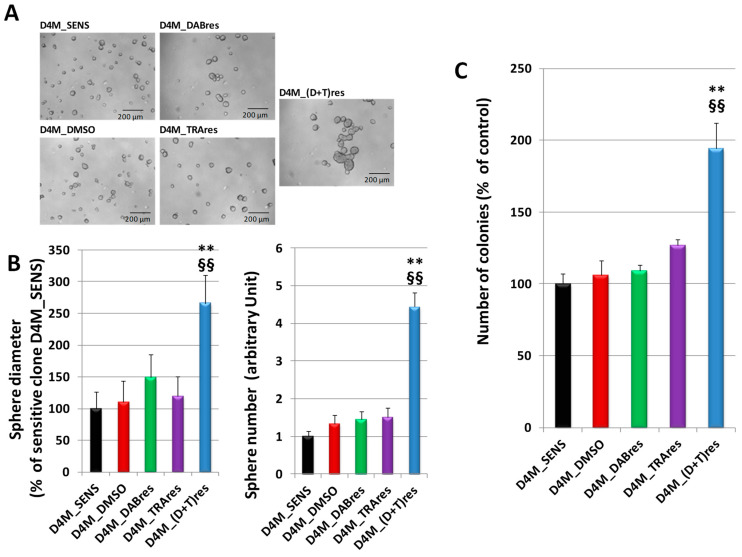
Anchorage-independent cell growth in untreated D4M_SENS, D4M_DMSO, D4M_DABres, D4M_TRAres, and D4M_(D+T)res. (**A**) Representative images of cell morphology obtained with a phase-contrast microscope in the sphere formation Assay. (**B**) Sphere diameter was expressed as the percent of spheres obtained in the sensitive clone D4M_SENS; sphere numbers greater than 50 μm were expressed as an arbitrary unit, normalized to the value obtained in the sensitive clone D4M_SENS. (**C**) Soft agar assay in untreated D4M_SENS, D4M_DMSO, D4M_DABres, D4M_TRAres, and D4M_(D+T)res. Colonies > 0.5 mm were counted using ImageJ software. Results were presented as the mean ± SD of triplicate samples from representative data of three independent experiments. ** *p* < 0.01 vs. D4M_SENS; §§ *p* < 0.01 vs. D4M_DMSO.

Sphere formation and soft agar assays were performed to assess the ability of cells to grow in an anchorage-independent manner. In Figure 2A, representative photographs of sphere formation of D4M_SENS, D4M_DMSO, D4M_DABres, D4M_TRAres, and D4M_(D+T)res cells are shown. Similar morphological pathways can be observed in all these subclones, with the exception of the D4M_(D+T)res cell line, where we observed bigger and greater numbers of tumor colonies than all the other cell sublines (Figure 2B). Similar results were obtained with the soft agar assay (Figure 2C), where the D4M_(D+T)res subclone showed a significantly enhanced cell growth.

#### 3.1.3. Apoptosis

**Figure 3:**
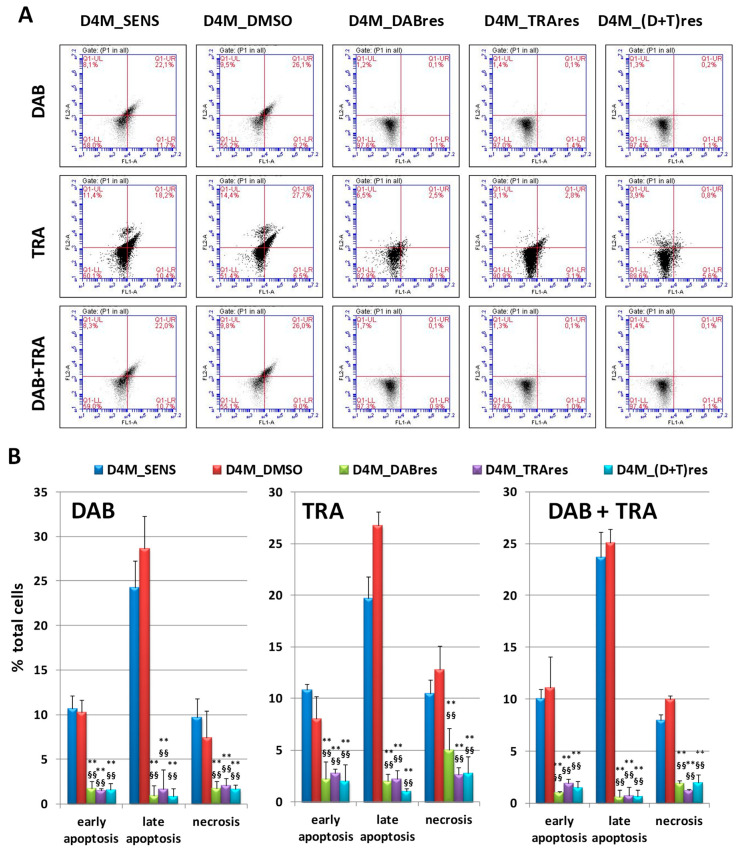
Apoptosis in D4M_SENS, D4M_DMSO, D4M_DABres, D4M_TRAres, and D4M_(D+T)res treated with 1.5 μM DAB, 36 nM TRA or combination (DAB + TRA). (**A**) The flow cytometry profiles of a representative experiment in Annexin V/IP-stained cells at 24 h are shown. Q1-LL = live (Annexin V−/PI−), Q1-LR = early stage of apoptosis (Annexin V+/PI−), Q1-UR = late stage of apoptosis (Annexin V+/PI+), and Q1-UL = necrosis (Annexin V−/PI+). (**B**) Histograms reporting cytofluorimetric analysis of Annexin V/PI staining in D4M treated sublines. Results of early and late apoptosis and necrosis were expressed as means ± SD of three independent experiments. ** *p* < 0.01 vs. D4M_SENS; §§ *p* < 0.01 vs. D4M_DMSO.

With the purpose of investigating the cell death role in the above-observed reduction of MTT values, Annexin-V/PI assay was performed in D4M_SENS, D4M_DMSO, D4M_DABres, D4M_TRAres, and D4M_(D+T)res cells treated with 1.5 μM DAB, 36 nM TRA or combined treatments for 24 h (Figure 3). From the cytofluorimetric analysis of the Annexin V/PI staining, we were able to discriminate necrotic (Annexin V−/PI+), early (Annexin V+/PI−), and late apoptotic (Annexin V+/PI+) cells. D4M_SENS and D4M_DMSO cells were affected by drug treatments. Indeed, high levels of Annexin V+, which identifies both early and late apoptosis, were observed in these sensitive clones, with values ranging between 30 and 40% of total cells. Conversely, the three resistant clones showed significantly lower levels of Annexin V+ cells, with values ranging between 1% and 5% of total cells. Thus, the three resistant clones showed similar results in all the experimental conditions.

#### 3.1.4. Cell Migration and Angiogenesis

**Figure 4:**
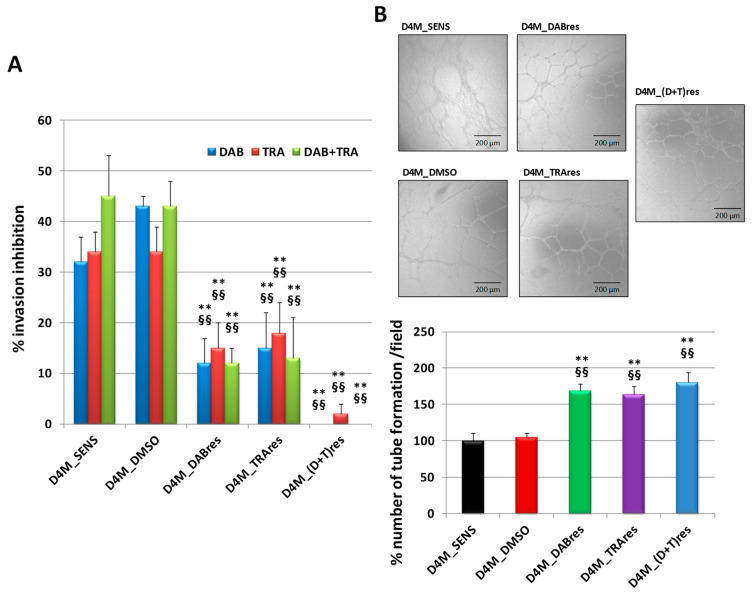
(**A**) Boyden chamber assay at 6 h in D4M_SENS, D4M_DMSO, D4M_DABres, D4M_TRAres, and D4M_(D+T)res sublines treated with 1.5 μM DAB, 36 nM TRA or combination (DAB + TRA). The results are expressed as a percentage of invasion inhibition, as the mean ± SD of five independent experiments. ** *p* < 0.01 vs. D4M_SENS; §§ *p* < 0.01 vs. D4M_DMSO. (**B**) Representative images of the tube formation assay on HUVECs after exposure to the conditioned media from untreated D4M_SENS, D4M_DMSO, D4M_DABres, D4M_TRAres, and D4M_(D+T)res subclones. Tube formation was photographed after 6 h incubation with these conditioned media and evaluated by counting the total number of tubes in three wells; three different experiments were performed. The results are illustrated in the histogram below. The data are the mean ± SD of three independent experiments ** *p* < 0.01 vs. D4M_SENS; §§ *p* < 0.01 vs. D4M_DMSO.

Tumor cell migration and angiogenesis, both essential components of the metastatic process, were investigated. The Transwell Boyden chamber assay in Matrigel was performed in D4M_SENS, D4M_DMSO, D4M_DABres, D4M_TRAres, and D4M_(D+T)res sublines. Cells were treated with 1.5 μM DAB, 36 nM TRA or combined treatments for 6 h, a non-toxic condition as demonstrated by MTT assays (data not shown). The results revealed that the invasion was remarkably inhibited by treatments in D4M_SENS and D4M_DMSO clones but not in the three resistant ones (Figure 4A). For the angiogenesis assay, the conditioned cell culture media of D4M_SENS, D4M_DMSO, D4M_DABres, D4M_TRAres, and D4M_(D+T)res subclones were collected to assess their ability to affect the tubule formation of HUVECs. The number of capillary-like structures formed by HUVECs was analyzed after 6 h of culture (Figure 4B). The conditioned cell culture media derived from the three resistant subclones induced a higher number of tubes with respect to that derived from D4M_SENS or D4M_DMSO control cells (19 ± 3 and 10 ± 1 number of tubes per field in D4M_SENS or D4M_DMSO, respectively). Moreover, the number of tubes obtained upon exposure to the conditioned cell culture media derived from the three resistant subclones was similar.

Collectively, these results allowed us to consider D4M_DABres, D4M_TRAres, and D4M_(D+T)res as good models of resistant melanoma cells toward targeted therapies. 

### 3.2. Analysis of the Redox Status and Nrf2 Expression in D4M Cell Lines Resistant to Dabrafenib, Trametinib, or Dual Resistance to Dabrafenib/Trametinib

**Figure 5:**
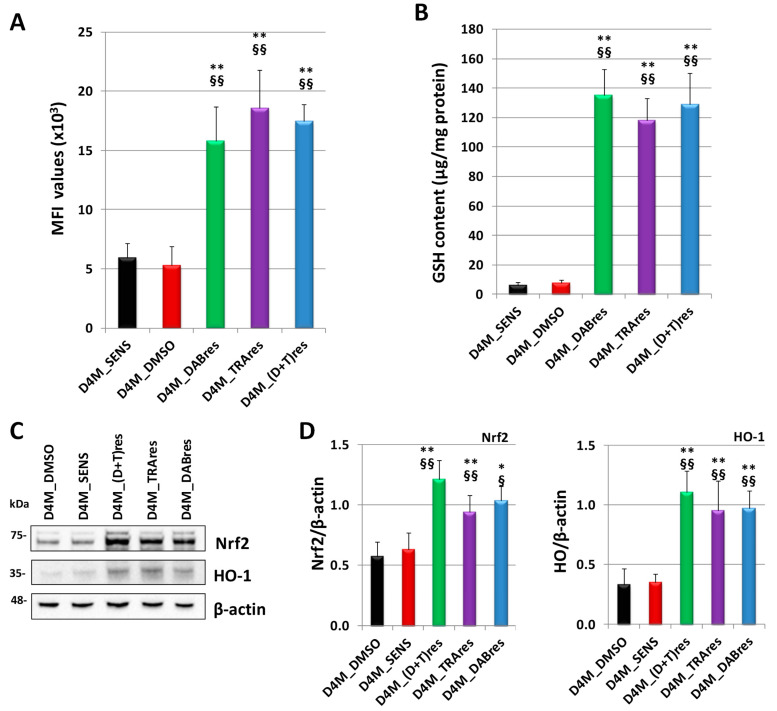
Nrf2 expression in D4M cell lines. (**A**) Intracellular oxidative stress levels in D4M_SENS, D4M_DMSO, D4M_DABres, D4M_TRAres, and D4M_(D+T)res untreated cells, measured by incubating cells with dichlorodihydrofluorescein diacetate (DCF-DA). The amount of fluorescent product (2,7-dichlorodihydrofluorescein, DCF) was measured by FACScan cytometer (Becton Dickinson Accuri). Bar graph showing median fluorescence intensity (MFI) values, expressed as means ± SD. ** *p* < 0.01 vs. D4M_SENS; §§ *p* < 0.01 vs. D4M_DMSO. (**B**) GSH level was evaluated in D4M_SENS, D4M_DMSO, D4M_DABres, D4M_TRAres, and D4M_(D+T)res untreated cells. Values are the mean ± SD of three separate evaluations. ** *p* < 0.01 vs. D4M_SENS; §§ *p* < 0.01 vs. D4M_DMSO. (**C**) Western blot analysis of Nrf2, and its target gene HO-1 in D4M_DMSO, D4M_SENS, D4M_(D+T)res, D4M_TRAres, and D4M_DABres untreated cells. (**D**) Densitometric analysis of the protein expression, normalized using the β-actin signal. Data are the mean ± SD of three independent experiments. * *p* < 0.05 and ** *p* < 0.01 vs. D4M_SENS; § *p* < 0.05 and §§ *p* < 0.01 vs. D4M_DMSO.

The redox status was evaluated by measuring the intracellular oxidative stress in the untreated D4M_SENS, D4M_DMSO, D4M_DABres, D4M_TRAres, and D4M_(D+T)res subclones. As shown in Figure 5A, this parameter was upregulated in all three resistant subclones [D4M_DABres, D4M_TRAres, and D4M_(D+T)res] when compared with sensitive cells (D4M_SENS, D4M_DMSO). However, the GSH content, the most important antioxidant molecule, was higher in the resistant subclones with respect to the sensitive sublines (Figure 5B). In accordance with the high level of GSH, Nrf2 protein expression was significantly higher in the resistant subclones with respect to the sensitive sublines, as well as the Nrf2 target HO-1 (Figure 5C,D). 

Oxidative stress, GSH, and Nrf2 content in the three resistant subcloned showed similar results. 

### 3.3. Nrf2 Affects YAP Expression in D4M Cell Lines Resistant to Dabrafenib, Trametinib, or Dual Resistance to Dabrafenib/Trametinib

**Figure 6:**
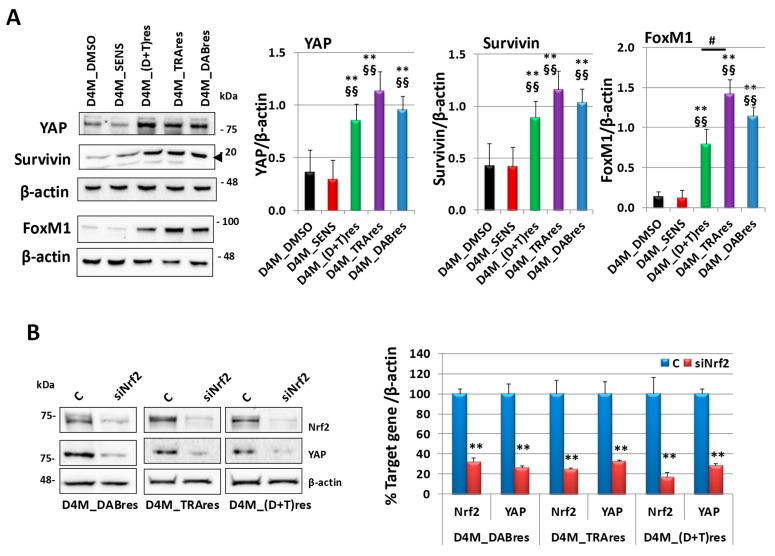
YAP expression and its regulation by Nrf2. (**A**) Western blot analysis of YAP and its target genes Survivin (the arrow is indicated the right band) and FoxM1 basal expression in D4M_DMSO, D4M_SENS, D4M_(D+T)res, D4M_TRAres, and D4M_DABres untreated cells. On the right densitometric analysis of the protein expressions, normalized using the β-actin signal. Data are the mean ± SD of three independent experiments. ** *p* < 0.01 vs. D4M_SENS; §§ *p* < 0.01 vs. D4M_DMSO; # *p* ≤ 0.05. (**B**) Western blot analysis of Nrf2 and YAP expressions in D4M-resistant cells in untreated control cells (C) or 24 h after the treatment with siRNA targeting Nfr2 (siNrf2). On the right is a densitometric analysis of protein expressions. Data were normalized using the β-actin signal and are indicated in the percentage of control values as the mean ± SD of three independent experiments. ** *p* ≤ 0.01 vs. C.

Previous results demonstrate the ability of Nrf2 to affect YAP expression in bladder [25] and pancreatic [26] cancer cells. To investigate a possible contribution of Nrf2 in regulating YAP expression in D4M cells, at first, we evaluated the basal expression of YAP and its targets Survivin and FoxM1 in D4M resistant cell lines. As shown in Figure 6A, analogously to Nrf2 expression, we observed an upregulation of YAP, Survivin, and FoxM1 in all three resistant subclones with respect to the sensitive ones. YAP and Survivin content was similar in all three resistant subclones; however, FoxM1 expression was significantly higher in D4M_TRAres with respect to D4M_(D+T)res but not D4M_DABres. 

Then, in the resistant subclones, we downregulated Nrf2 with a specific siRNA, demonstrating that the Nrf2 inhibition elicited a reduction of YAP expression (Figure 6B). A negative control siRNA was used to exclude non-specific effects in the RNA interference (RNAi) experiments. As shown in Figure S1, it did not affect Nrf2 or YAP expression in all three resistant subclones.

### 3.4. Mechanism of Nrf2 Activity Regulation in D4M Cell Lines Resistant to Dabrafenib, Trametinib, or Dual RESISTANCE to Dabrafenib/Trametinib

**Figure 7:**
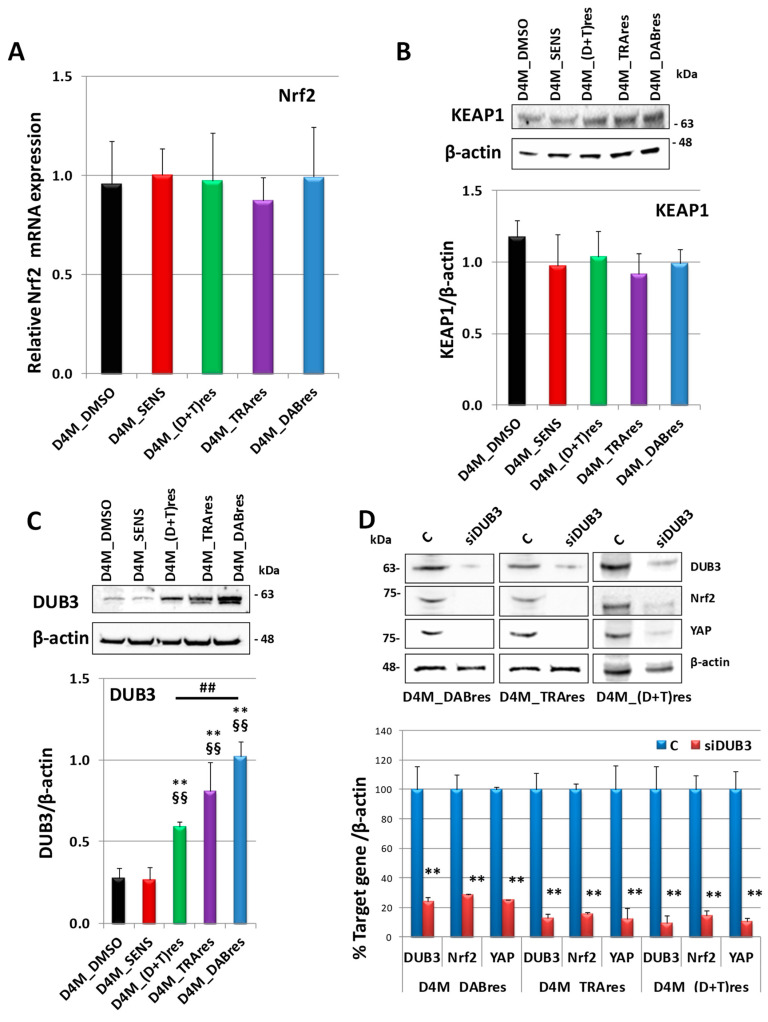
Nrf2 expression regulation. (**A**) Nrf2 mRNA expression in D4M_DMSO, D4M_SENS, D4M_(D+T)res, D4M_TRAres, and D4M_DABres untreated cells. mRNA expression was evaluated by qRT-PCR in triplicate. Abelson (Abl) gene was utilized as a housekeeping control. Results showing a discrepancy greater than one cycle threshold in one of the wells were excluded. The results were analyzed using the ΔΔCt method. (**B**) Western blot analysis of KEAP1 in D4M_DMSO, D4M_SENS, D4M_(D+T)res, D4M_TRAres, and D4M_DABres untreated cells. Below is a densitometric analysis of the protein expression, normalized using the β-actin signal. Data are the mean ± SD of three independent experiments. (**C**) Western blot analysis of DUB3 in D4M_DMSO, D4M_SENS, D4M_(D+T)res, D4M_TRAres, and D4M_DABres untreated cells. Below is a densitometric analysis of the protein expression, normalized using the β-actin signal. Data are the mean ± SD of three independent experiments. ** *p* < 0.01 vs. D4M_SENS; §§ *p* < 0.01 vs. D4M_DMSO. ## *p* < 0.01. (**D**) Western blot analysis of DUB3, Nrf2, and YAP expressions in D4M resistant cells in untreated control cells (C) or after 24 h from the treatment with siRNA targeting DUB3 (siDUB3). On the right is a densitometric analysis of protein expressions. Data were normalized using the β-actin signal and are indicated in the percentage of control values as the mean ± SD of three independent experiments. ** *p* ≤ 0.01 vs. C.

In the attempt to investigate the mechanisms of Nrf2 protein upregulation in the resistant subclones, we started by quantifying mRNA Nrf2 by qRT-PCR (Figure 7A). Our results demonstrated there was no correspondence between mRNA and protein expressions, suggesting a post-translational control of Nrf2 expression.

To deeply investigate the mechanisms leading to the increased Nrf2 protein expression, we analyzed the intracellular levels of KEAP1, which binds and drives Nrf2 to proteasomal degradation. However, as shown in Figure 7B, its content was not changed in resistant subclones with respect to the sensitive sublines, suggesting that the reduction of Nrf2 protein did not depend on KEAP1 upregulation.

Given the role of DUB3 in controlling the Nrf2 protein level, its expression was analyzed in the melanoma cell lines. As shown in Figure 7C, we observed an upregulation in all three resistant subclones with respect to the sensitive ones. Of note, DUB3 expression in the dual resistant subclone (D4M_(D+T)res was significantly lower than the resistant clone D4M_Dres but not D4M_Tres. Moreover, the downregulation of DUB3 with a specific siRNA elicited not only a reduction of Nrf2 but also of YAP protein expression (Figure 7D). The specificity of the RNAi experiments was confirmed by treating cells with a negative control siRNA. As shown in Figure S1, siNeg treatment did not affect any of the target genes studied in all three resistant subclones.

### 3.5. Inhibition of Nrf2 or DUB3 Expression Sensitizes Resistant Melanoma D4M to Targeted Therapies

**Figure 8:**
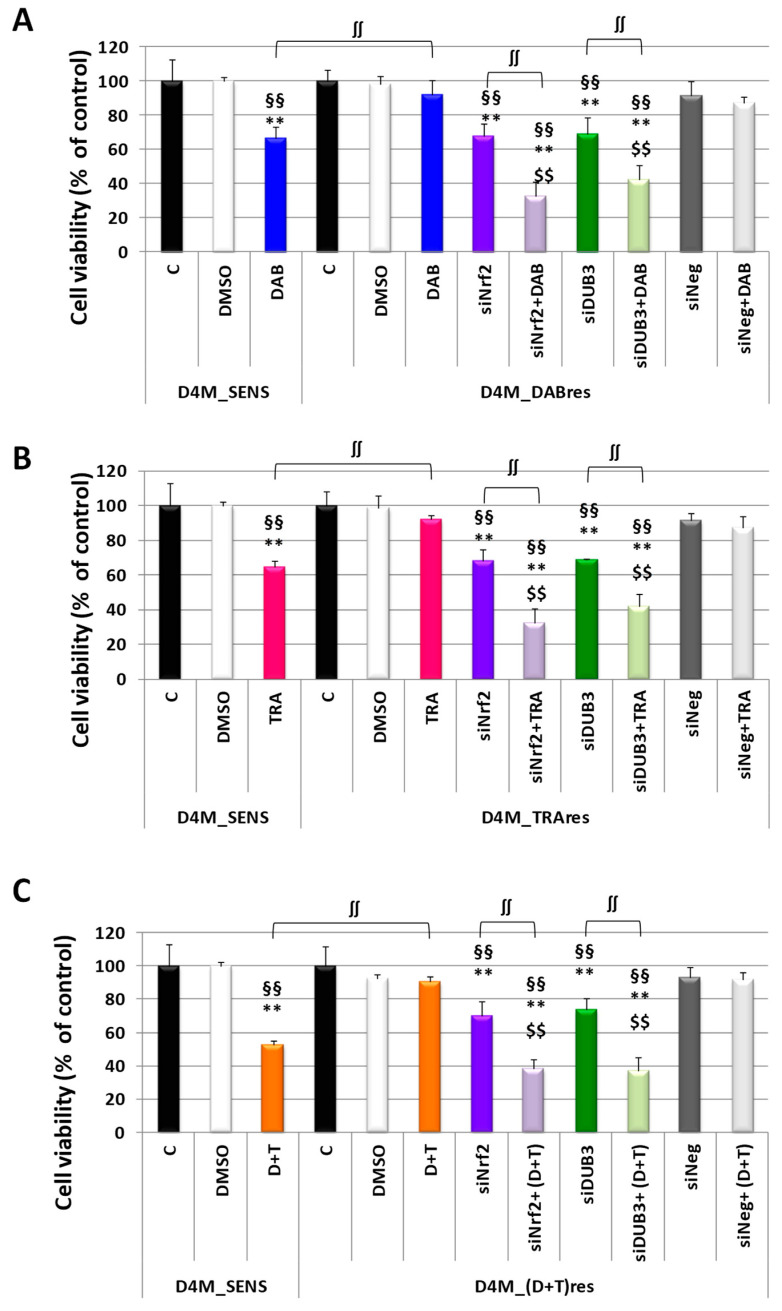
Viability (MTT assay) in D4M_SENS or resistant subclones treated with specific siRNAs targeting Nrf2 (siNrf2) or DUB3 (siDUB3). (**A**) Viability in untreated D4M_SENS (Control, C) or treated with DMSO or 1.5 μM DAB; viability in untreated D4M_DABres cells (Control, C) or treated with DMSO (DMSO), 1.5 μM DAB (DAB), siNrf2, siNrf2 plus 1.5 μM DAB (siNrf2+DAB), siDUB3, siDUB3 plus 1.5 μM DAB (siDUB3+DAB), siNeg, siNeg plus 1.5 μM DAB (siNeg+DAB). Results are expressed as a percent of control and are the mean ± SD of three separate experiments. ** *p* < 0.01 vs. respective Control untreated cells; §§ *p* < 0.01 vs. respective DMSO treated cells; $$ *p* < 0.01 vs. D4M_DABres or D4M_SENS cells treated with DAB; ∫∫ *p* < 0.01. (**B**) Viability in untreated D4M_SENS (Control, C) or treated with DMSO or 36 nM TRA; viability in untreated D4M_TRAres cells (Control, C) or treated with DMSO (DMSO), 36 nM TRA (TRA), siNrf2, siNrf2 plus 36 nM TRA (siNrf2+tra), siDUB3, siDUB3 plus 36 nM TRA (siDUB3+TRA), siNeg, siNeg plus 36 nM TRA (siNeg+TRA). Results are expressed as a percent of control and are the mean ± SD of three separate experiments. ** *p* < 0.01 vs. respective Control untreated cells; §§ *p* < 0.01 vs. respective DMSO treated cells; $$ *p* < 0.01 vs. D4M_TRAres or D4M_SENS cells treated with 36 nM TRA; ∫∫ *p* < 0.01. (**C**) Viability in untreated D4M_SENS (Control, C) or treated with DMSO or 1.5 μM DAB plus 36 nM TRA combined treatments (D+T); viability in untreated D4M_(D+T)res cells (Control, C) or treated with DMSO (DMSO), 1.5 μM DAB plus 36 nM TRA combined treatments (D+T), siNrf2, siNrf2 plus combined treatment (siNrf2+ (D+T), siDUB3, siDUB3 plus plus combined treatment (siNrf2+ (D+T), siNneg, siNeg plus combined treatment (siNrf2+ (D+T). Results are expressed as a percent of control and are the mean ± SD of three separate experiments. ** *p* < 0.01 vs. respective Control untreated cells; §§ *p* < 0.01 vs. respective DMSO treated cells; $$ *p* < 0.01 vs. D4M_(D+T)res cells or D4M_SENS cells treated with 1.5 μM DAB plus 36 nM TRA combined treatments (D+T); ∫∫ *p* < 0.01.

The high Nrf2 expression in resistant D4M melanoma cell lines led us to investigate whether its inhibition could sensitize them to targeted therapies. Interestingly, the Nrf2 inhibition alone, obtained with a specific siRNA, elicited a significant viability inhibition in D4M_DABres cells with respect to untreated or DMSO-treated cells at 24 h; moreover, at the same time, the Nrf2 inhibition greater sensitized the D4M_DABres cell line to DAB (Figure 8A). Of note, D4M_DABres treated with siNrf2 plus DAB were significantly more inhibited not only toward the same cells treated with siNrf2 or DAB alone but also with respect to D4M_SENS cells treated with DAB (Figure 8A). Similar results were obtained in D4M_TRAres cells treated with siNrf2 plus TRA (Figure 8B) or in D4M_(D+T)res treated with siNrf2 plus DAB+TRA (D+T) (Figure 8C).

Since DUB3 expression was found to be higher in resistant subclones and this deubiquitinase was able to control Nrf2 expression, we decided to investigate the effects of DUB3 inhibition in these cells. As shown in Figure 8, similarly to Nrf2 inhibition, the DUB3 inhibition alone, obtained with a specific siRNA, elicited a significant viability inhibition in all three resistant subclones with respect to untreated or DMSO-treated cells at 24 h; moreover, at the same time, the DUB3 inhibition greater sensitized all these resistant subclones to targeted drugs. SiNeg treatment did not affect cell viability and did not sensitize resistant cell lines to targeted drugs.

### 3.6. Nrf2 and YAP Expression in A375 Cell Line Resistant to Dabrafenib

**Figure 9:**
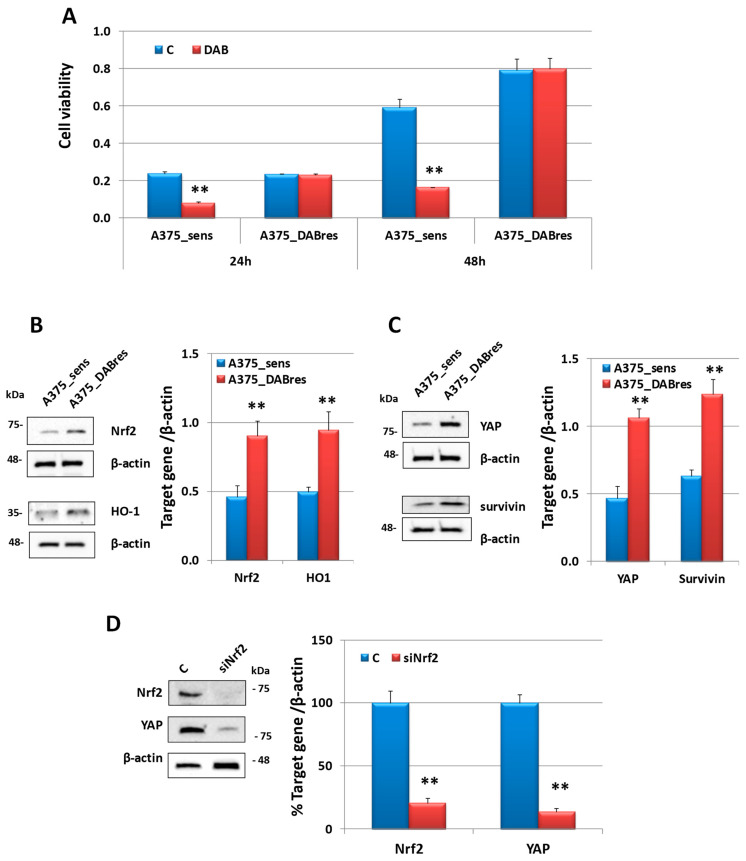
Analysis in A375_sens and A375_DABres human melanoma cell lines. (**A**) Viability (MTT assay) in A375_sens and A375_DABres untreated (control, C) or treated with dabrafenib 200 nM (DAB). Results are the mean ± SD of three separate experiments. ** *p* < 0.01 vs. A375_sens. (**B**) Western blot analysis of Nrf2 and its target gene HO-1 in A375_sens and A375_DABres untreated cells. On the right is a densitometric analysis of the protein expressions, normalized using the β-actin signal. Data are the mean ± SD of three independent experiments. ** *p* < 0.01 vs. A375_sens. (**C**) Western blot analysis of YAP and its target gene Survivin in A375_sens and A375_DABres untreated cells. On the right is a densitometric analysis of the protein expressions, normalized using the β-actin signal. Data are the mean ± SD of three independent experiments. ** *p* < 0.01 vs. A375_sens. (**D**) Western blot analysis of Nrf2 and YAP expressions in untreated A375_DABres untreated control cells (C) or after 24 h from the treatment with siRNA targeting Nfr2 (siNrf2). On the right is a densitometric analysis of protein expressions. Data were normalized using the β-actin signal and are indicated in the percentage of control values as the mean ± SD of three independent experiments. ** *p* ≤ 0.01 vs. C.

To verify the role of Nrf2 in another cell line resistant to targeted therapy, we analyzed the Nrf2 protein expression in an A375 human melanoma cell line sensitive (A375_sens) or resistant to dabrafenib (A375_DABres), obtained in the laboratories of Prof. Caputo [38]. After confirming the resistance to DAB treatments (Figure 9A) with an MTT assay, we demonstrated that Nrf2 protein expression and its target HO-1 were higher in the resistant subclone (Figure 9B). Moreover, we also confirmed in this cell line that the basal expression of YAP and its target Survivin was upregulated in the resistant subclone (Figure 9C) and that the inhibition of Nrf2 with a specific siRNA elicited a reduction of YAP expression (Figure 9D). A negative control siRNA was used to exclude non-specific effects in the RNA interference (RNAi) experiments. As shown in Figure S1, it did not affect Nrf2 or YAP expression in the A375_DABres cell line.

### 3.7. Mechanisms of Nrf2 Gene Expression Control in an A375 Cell Line Resistant to Dabrafenib

**Figure 10:**
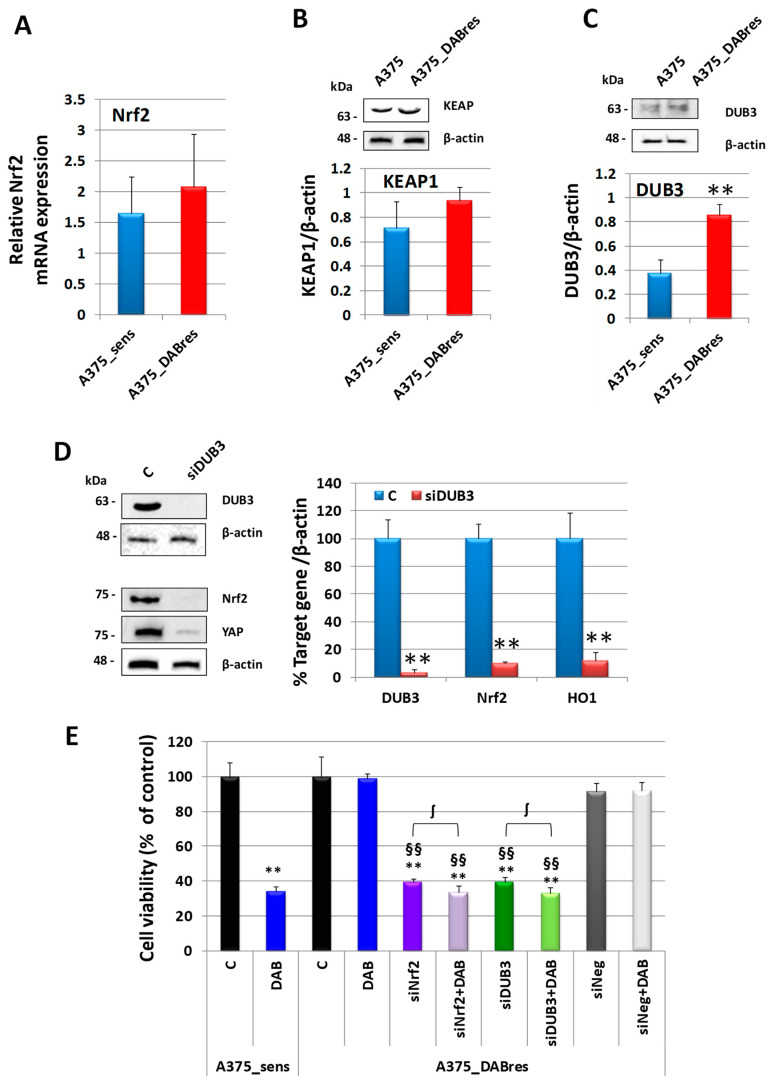
Nrf2 expression regulation in A375 cells. (**A**) Nrf2 mRNA expression in A375_sens and A375_DABres untreated cells. mRNA expression was evaluated by qRT-PCR in triplicate. Abelson (Abl) gene was utilized as a housekeeping control. Results showing a discrepancy greater than one cycle threshold in one of the wells were excluded. The results were analyzed using the ΔΔCt method. (**B**) Western blot analysis of KEAP1 in A375_sens and A375_DABres untreated cells. Below is a densitometric analysis of the protein expression, normalized using the β-actin signal. Data are the mean ± SD of three independent experiments. (**C**) Western blot analysis of DUB3 in A375_sens and A375_DABres untreated cells. Below is a densitometric analysis of the protein expression, normalized using the β-actin signal. Data are the mean ± SD of three independent experiments. ** *p* < 0.01 vs. A375_sens. (**D**) Western blot analysis of DUB3, Nrf2, and YAP expressions in A375_DABres untreated control cells (C) or after 24 h from the treatment with siRNA targeting DUB3 (siDUB3). On the right is a densitometric analysis of protein expressions. Data were normalized using the β-actin signal and are indicated in the percentage of control values as the mean ± SD of three independent experiments. ** *p* ≤ 0.01 vs. C. (**E**) Viability in untreated A375_sens (Control, C) or treated with 200 nM DAB; viability in A375_DABres cell untreated (control, C) or treated with siNrf2, siNrf2 plus 200 nM DAB, siDUB3, siDUB3 plus 200 nM DAB, siNneg, siNeg plus 200 nM DAB. Results are expressed as percent of control and are the mean ± SD of three separate experiments. ** *p* < 0.01 vs. respective Control untreated cells; §§ *p* < 0.01 vs. respective 200 nM DAB treated cells; ∫ *p* < 0.05.

In agreement with the results obtained in D4M resistant cells, we demonstrated in A375 cell lines that there was not any correspondence between mRNA and protein expressions, suggesting a post-translational control of Nrf2 expression (Figure 10A). Moreover, KEAP1 was not affected (Figure 10B), while the expression of DUB3 was upregulated in the resistant cell line A375_DABres (Figure 10C). Finally, similarly to that observed in D4M resistant cell lines, the downregulation of DUB3 with a specific siRNA elicited not only a reduction of Nrf2 but also YAP protein expression (Figure 10D). The specificity of the RNAi experiments was confirmed by treating cells with a negative control siRNA. As shown in Figure S1, siNeg treatment did not affect any of the target genes studied in the A375_DABres cell line.

### 3.8. Inhibition of Nrf2 or DUB3 Expression Sensitizes Resistant A375 Melanoma Cells to Targeted Therapy

Similar to what was observed in D4M resistant cells, the inhibition of Nrf2 or DUB3 in A375_DABres cells can sensitize cells to DAB treatment. Of note, the Nrf2 inhibition alone elicited a significant viability inhibition in A375_DABres cells with respect to untreated control (Figure 10E).

## 4. Discussion

In the present study, we demonstrated a key role of Nrf2 in melanoma BRAFi/MEKi resistance. To conduct our research, we took advantage of BRAF-mutated melanoma-resistant subclones derived from murine D4M cells, here obtained and characterized, and from the human A375 cell line previously established [38]. 

We observed that all the resistant subclones were significantly less sensitive to the drug treatments with respect to sensitive cell lines. Interestingly, the D4M subclones with DAB single-agent resistance were also resistant to TRA and vice versa; moreover, the D4M subclone with dual resistance (DAB+TRA) was resistant not only to the combined treatment but also to the single ones. These findings can be explained by considering that these two drugs are affecting the same signaling pathway, the MAPK cascade.

All three D4M resistant subclones can evade BRAFi/MEKi-induced apoptosis, have an enhanced tumor invasion ability, and exhibit high capability in inducing angiogenesis. Moreover, dual resistance D4M cells, but not D4M_Tres or D4M_Dres single resistant clones, showed an enhanced anchorage-independent growth. This ability is considered a hallmark of carcinogenesis, and it is also able to identify tumors with metastatic potential [44]. Thus, we can suggest that acquiring dual resistance can induce a more aggressive phenotype, possibly revealed with further in vivo experiments which can allow the evaluation of metastasis formation. Collectively, we were able to demonstrate that the acquisition of BRAFi/MEKi resistance promotes an aggressive phenotype, prone to enhanced cancer growth, invasion, and angiogenesis, all being prerequisites for metastasis formation and in agreement with clinical findings [45]. These results allowed us to consider D4M_DABres, D4M_TRAres, and D4M_(D+T)res as good models of resistant melanoma cells toward targeted therapies. The choice of a mouse model also opens possibilities for future in vivo studies in an immunocompetent animal. This possibility is attractive since it has been shown that the tumor-infiltrating immune cells can participate in regulating BRAFi resistance [46].

Accumulating evidence indicated that cancer cells, including melanoma, exhibit high levels of ROS/RNS compared to physiological conditions [12,47]. According to literature data, both internal and environmental mechanisms have been shown to be involved in the ROS/RNS increase. ROS production is enhanced as an internal mechanism as a consequence of oncogene activation, tumor suppressor loss, hypoxia, or reprogrammed metabolisms [48]. As external stimuli, cigarette smoke and ultraviolet (UV) radiation are the main reasons for the oxidative stress increase [49,50]. However, in comparison with other solid cancers, ROS production is particularly elevated in melanoma due to the natural exposure to exogenous UV radiation and the biochemical pathway leading to melanin synthesis, both eliciting ROS production [50]. Interestingly, the presence of the BRAF mutation was found to be involved in regulating oxidative stress since it can be associated with enhanced glycolytic metabolism and lowering mitochondrial oxidative phosphorylation (OXPHOS), one of the main sources of ROS production; however, BRAFi/MEKi-resistant melanoma cells show an enhancement of mitochondrial biogenesis, activity, and content, leading to a further increase in mitochondrial ROS production [51,52]. The switch from glycolysis toward OXPHOS can be considered an adaptative metabolic rewiring that allows melanoma cells to produce sufficient ATP levels to survive, thus counteracting the inhibition of glycolysis induced by BRAFi/MEKi treatment [53]. In agreement with these findings, we demonstrated that all the D4M BRAFi/MEKi-resistant subclones exhibited an enhanced oxidative stress level. An increase in mitochondrial respiration and subsequent ROS enhancement was already demonstrated in several human melanoma cells, including A375 cells, resistant to BRAFi. In particular, both vemurafenib-resistant, DAB-resistant, and DAB/TRA double-resistant A375 subclones exhibited an increase in mitochondrial respiration and ROS production [54,55,56,57]. 

In addition to the above-mentioned metabolic rewiring, treatment with BRAFi or MEKi alone or in combination can increase per se oxidative stress within the cancer cell by upregulating ROS production [58,59]. Thus, to cope with this huge amount of free radical production, BRAFi/MEKi-resistant melanoma cells enhance their antioxidant systems to survive under oxidative stress. As demonstrated here, the GSH level increased in all the D4M BRAFi/MEKi-resistant subclones. The upregulation of GSH content was previously demonstrated in vemurafenib-resistant A375 cells, which are also resistant to DAB, TRA, or a combination [55]. 

Consistent with the GSH higher level, we demonstrated an increase in Nrf2 protein expression in all resistant lines used in our study. In particular, Nrf2 was upregulated in both D4M and A375 cells resistant to DAB. This result agrees with results presented by Khamari and collaborators [55], who demonstrated an upregulation in melanoma cells resistant to the BRAFi vemurafenib, obtained in vivo, by continuously treating SCID mice engrafted with A375 cells with the drug. Moreover, our data demonstrated that Nrf2 is also elevated in TRA-resistant cells and in dual-resistance DAB/TRA. Thus, it appears that Nrf2 is not only characteristic of the resistance induction to BRAF inhibitor drugs but, more generally, it plays a role in resistance to MAPK pathway inhibitors. 

Consistent with the increase of Nrf2, its HO-1 downstream gene was found to be upregulated in all the cell lines resistant to targeted therapy. The HO-1 enzyme can degrade the pro-oxidant free heme molecule into CO, ferrous iron, and biliverdin, which is quickly transformed into bilirubin. The end products of HO-1 have antioxidant activities, and HO-1 overexpression has been found in various tumor types, facilitating tumor growth and drug resistance [60]. In melanoma cells, overexpression of HO-1 leads to enhanced tumor growth, angiogenesis, and resistance to anticancer treatment [61]. Moreover, HO-1 is associated with poor clinical outcomes of uveal melanoma [62]. Interestingly, HO-1 was found to be upregulated in a series of BRAF-mutated melanoma cells exposed for 24 h to vemurafenib [63]. 

As reported in the results section, we demonstrated that all the resistant cell lines used in this study showed an increase in the levels of YAP, one of the most studied oncogenes involved in initiation, proliferation, and metastasis of tumor cells as well as chemoresistance [64]. Its target Survivin was highly expressed in advanced melanoma disease [65], as well as FoxM1, which plays an important role in melanoma progression and chemoresistance [66]. Our results agree with the literature data, highlighting the role of YAP in targeted resistance. Indeed, it was demonstrated that the activation of YAP occurred in melanoma cells resistant to vemurafenib [67] and that the inhibition of YAP function overcame BRAFi resistance in melanoma cancer stem cells [68]. Here we present novel Nrf2 abilities since we demonstrated that the upregulation of YAP was consistent with that of Nrf2 and that cooperation between these two factors exists in melanoma cells also [25]. Indeed, Nrf2 downregulation by RNAi led to YAP protein inhibition in all the BRAFi/MEKi-resistant melanoma cell lines used in this study. Further studies are needed to better elucidate the precise mechanism. Interestingly, we can suggest the contribution of the YAP target FoxM1 in sustaining Nrf2 transcription since it has been demonstrated that Nrf2 can be transcriptionally activated by FoxM1 [69]. Collectively, these data suggest that adaptative redox conditions are characterized not only by an increase in the expression of a wide range of cytoprotective and antioxidant genes but also by enhanced activity in other significant molecular pathways with well-recognized involvement in cancer progression and chemoresistance. 

It has been demonstrated that BRAF mutations resulted in increased mRNA Nrf2 levels [70]; however, accumulating evidence showed that post-translational mechanisms regulating Nrf2 protein content mainly occur in melanoma [33]. Our data confirmed these findings since no significant differences were seen in the qRT-PCR performed, comparing the three D4M resistant subclones with the sensitive ones. Although KEAP1 is one of the main regulators of the Nrf2 protein stability, since its binding with Nrf2 leads to ubiquitination and proteasomal degradation of Nrf2 [71], KEAP1 expression was not changed in our experimental setting. We cannot completely rule out the contribution of KEAP1 since inactivating mutations of this inhibitor may have been caused by continued drug treatment. However, we can certainly exclude the intervention of post-translational mechanisms leading to a decrease in KEAP1 content, such as the p62 Nrf2-KEAP2 disruptor, or the dysregulation of microRNA targeting KEAP1, since the KEAP1 protein content remains unchanged in all melanoma clones studied here.

Recently, the contribution of DUB3 in controlling Nrf2 protein stability in colorectal cancer and resistant PANC-1 pancreatic cells has been shown [26,31]. Approximately 100 putative DUB enzymes can regulate protein stability and thereby control several important cellular processes. They can be grouped into five main classes: ubiquitin-specific proteases (USPs), the cysteine proteases ubiquitin C-terminal hydrolases (UCHs), ovarian tumor proteases (OTUs), the metalloproteases JAB1/MPN/MOV34 (JAMM), and the Machado-Joseph domain proteases (MJDs) [72]. Recently, the emerging role of DUBs in melanoma progression and the development of therapeutic resistance were highlighted [36]. DUB3 belongs to the USP17 gene family; it is highly expressed in cancer cell lines and has an established role in tumor proliferation [73]. Here we reported an upregulation of DUB3 in all the resistant cells used in this study with respect to the sensitive clones. Notably, DUB3 expression in D4M_(D+T)res was significantly lower than in the resistant clone D4M_Dres but not D4M_Tres. Further studies are needed to clarify this difference. For instance, assessing the DUB3 mRNA transcriptional rate or the stability of DUB3 protein in all three subclones can be decisive in explaining the observed differences. Unsurprisingly, the inhibition of DUB3 led to an inhibition of Nrf2 in all the BRAFi/MEKi-resistant cells used in this study. More interestingly, it also resulted in significant inhibition of YAP expression. We can postulate that the inhibition of Nrf2 expression, through DUB3 silencing, was able to reduce YAP expression, as previously demonstrated by Nrf2 silencing. However, we cannot exclude that DUB3 could directly affect YAP expression. Moreover, these mechanisms were not limited to a single experimental model, but they were also demonstrated in A375 human melanoma cell lines. Importantly, either through the inhibition of Nrf2 directly or through the inhibition of DUB3, resistant subclones of melanoma were more responsive to the targeted therapies. Not only the inhibition of Nrf2 and its targets but also that of YAP and its signaling may contribute to the increased sensitivity. 

## 5. Conclusions

On the whole, our results demonstrated that high levels of Nrf2 are involved in maintaining resistance to BRAFi/MEKi therapies in melanoma. Moreover, Nrf2 appears to assume a central role in the control not only of its signaling but also that of Hippo, a very important dysregulated pathway in cancer. Furthermore, the possibility that the transcription factor Nrf2 can also indirectly affect the expression of other transcription factors or transcriptional coactivators, such as FoxM1 and YAP, greatly increases the number of genes it controls, thus extending the possibilities of affecting a multitude of cellular functions. The antiproliferative effects of direct inhibition of Nrf2 or indirectly, through down-regulation of DUB3, are superimposable. This greatly increases the possibilities for therapeutic intervention and highlights the interest in DUBs as druggable targets in melanoma. 

## Data Availability

The data presented in this study are available in the article and Supplementary Materials.

## Supplementary Materials

The following supporting information can be downloaded at: https://www.mdpi.com/article/10.3390/antiox12061313/s1, Figure S1: Effect of negative control siRNA treatment on Nrf2, YAP, and DUB3 expressions.
