# Supplementary material for: Nrf2 as a Therapeutic Target in the Resistance to Targeted Therapies in Melanoma
Source: Antioxidants (Basel). 2023 Jun 20;12(6):1313. doi: 10.3390/antiox12061313 (PMC10294952; doi:10.3390/antiox12061313)
Supplement: Supplementary file 1 [file antioxidants-12-01313-s001.zip › antioxidants-2443688-supplementary.pdf]

Supplementary data

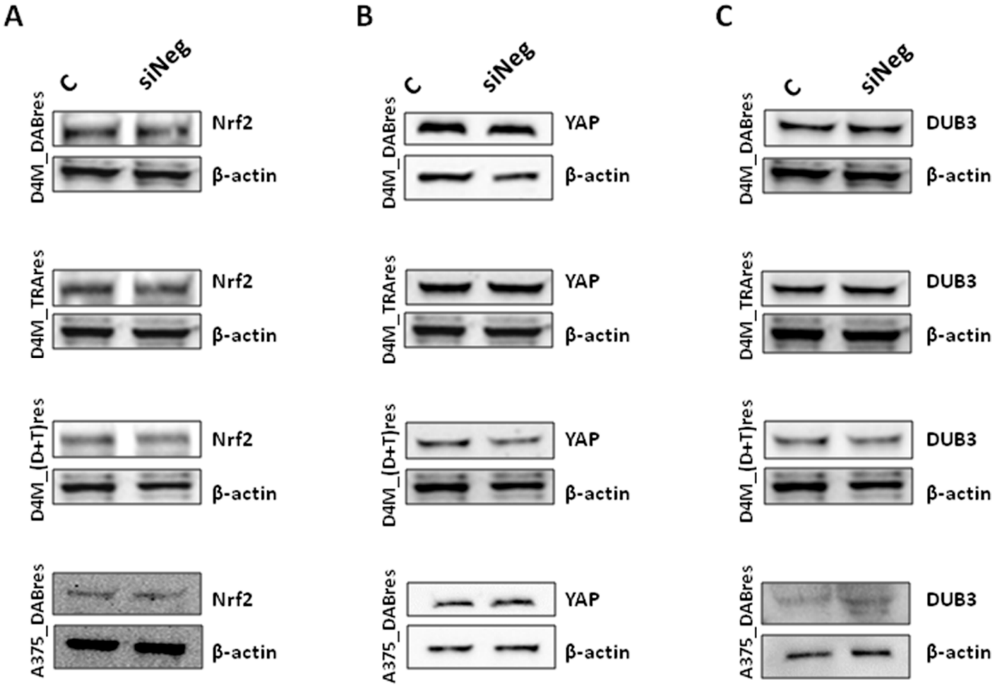

Figure S1 Effect of negative control siRNA (siNeg) treatment on Nrf2 (**panel A**), YAP (**Panel B**), and DUB3 (**Panel C**) expressions in D4M and A375 resistant subclones. Western blot analysis in D4M_DABres, D4M_TRAres, and D4M_(D+T)res untreated control cells (C) or after 24 h from the treatment with siNeg. Equal protein loading was confirmed by analysis of relative β-actin expressions.
